# Changes and tracking of fruit, vegetables and sugar-sweetened beverages intake from 18 months to 7 years in the Norwegian mother and child cohort study

**DOI:** 10.1186/1471-2458-13-793

**Published:** 2013-08-30

**Authors:** Mona Bjelland, Anne Lise Brantsæter, Margaretha Haugen, Helle Margrete Meltzer, Wenche Nystad, Lene Frost Andersen

**Affiliations:** 1 Department of Nutrition, University of Oslo, P.O. Box 1046 Blindern, NO-0316 Oslo, Norway; 2 Division of Environmental Medicine, Norwegian Institute of Public Health, Oslo, Norway; 3 Division of Epidemiology, Norwegian Institute of Public Health, Oslo, Norway

**Keywords:** Children, Tracking, Diet, MoBa

## Abstract

**Background:**

A few studies have investigated tracking of dietary patterns or nutrient intake in pre-school children, but no studies have been identified examining tracking of sugar-sweetened beverages (SSB), fruit and vegetable intakes in early childhood (1–7 year olds). The purpose of this study was to investigate changes and tracking of intakes of fruit, vegetables and SSB, and association between maternal education and dietary tracking, from 18 months to 7 years of age.

**Methods:**

Longitudinal data from the nation-wide Norwegian Mother and Child Cohort Study, conducted by the Norwegian Institute of Public Health were used, including 9 025 children participating at three time points (18 months, 36 months and 7 years). Frequencies of fruit, vegetables and SSB were assessed by questionnaire. Slightly different questions were used at each time point to collect information about intake. Maternal education was categorized into ≤ 12 years, 13–16 years, ≥ 17 years. Cross-tabulation, Spearman’s rho and multinomial logistic regression were used for assessing change, tracking and differences by maternal education.

**Results:**

Analyses by gender indicated largest changes for intake of fruit and SSB from age 18 months to 7 years. Fair to moderate tracking coefficients (Spearman’s rho = 0.23-0.46) for intake of fruit, vegetables and SSB were found and children assigned to low, medium and high frequency of consumption at 18 months continued to be in the same group at age 36 months and 7 years. Children of mothers with low education consumed fruit and vegetables less often and SSB more often compared to children of mothers with high education at 18 months of age. Children with higher educated mothers had lower odds for increasing fruit intake or decreasing SSB intake, compared to children with lower educated mothers showing a stable intake.

**Conclusions:**

The tracking coefficients for intakes were fair to moderate and differences in intakes according to maternal education were found already at age 18 months. This suggests that promotion of healthy dietary behaviours at an early age is important to prevent unfavourable dietary behaviours later in childhood. Moreover, it seems important to target mothers in nutrition interventions for improving dietary habits among children.

## Background

The intake of fruit and vegetables is considered an important part of a healthy lifestyle. An adequate intake of fruit and vegetables may reduce energy density, prolong satiety, increase fibre intake and decrease overall energy intake [1-3]. The opposite is the case for sugar-sweetened beverages (SSB); several recent reviews report a statistically significant association between consumption of SSB and BMI/weight/adiposity/weight gain based on a combination of cross-sectional, prospective and intervention studies [4-8]. The national recommendations in Norway from 1996 to 2011 was to eat at least five portions of fruit and vegetables a day; three servings of vegetables and two servings of fruit [9]. Earlier Norwegian studies have observed a high intake of energy from added sugar and SSB (such as carbonated soft drinks and/or cordials) and a low intake of fruit and vegetables among children and adolescents [2,10-14]. A small decrease in frequency of fruit and vegetable intake has been reported among Norwegian 11–13 year olds in the period 2001 to 2008 [15]. A decrease was also observed for frequency in intake of SSB in the same age group and time period [16].

Parental education, one indicator of a family’s socio-economic status, appears to be an important determinant of dietary intake in children [17]. Several studies covering the toddler and preschool years find that children of the most educated mothers have the most healthy diets [18-24]. The education level has important influence on maternal nutrition knowledge, which is associated with compliance to dietary guidelines in young children and mediates the association between socioeconomic status and diet quality in mothers [25,26].

Tracking can be defined as the stability of health-related behaviours over time or as stability in rank at the group level [27]. A few studies have investigated tracking of dietary patterns or nutrient intake in pre-school children [22,28-31]. To our knowledge no studies have examined tracking of SSB, fruit and vegetable intakes in early childhood (1–7 year olds). In a public health perspective it is important to target nutrition education and interventions to groups with poor dietary habits as early as possibly in a life-course [21]. Furthermore, exploring when, how and why dietary changes occur over time is critical to being able to develop strategies for interventions to ensure that children have the best nutritional start to life [24,32].

The present study aimed to investigate the changes and tracking in intakes of fruit, vegetables and SSB in a group of Norwegian children, from 18 months to the age of 7 years. Furthermore, a second aim was to examine the association between maternal education and dietary tracking in the same group of young children.

## Methods

The Norwegian Mother and Child Cohort Study (MoBa) is a prospective population-based pregnancy cohort study conducted by the Norwegian Institute of Public Health [33]. Participants were recruited from all over Norway from 1999–2008, and 38.5% of invited women consented to participate. This study used version 6 of the quality-assured data files made available for research in 2011. Informed consent was obtained from each participant upon recruitment. The Regional Committee for Medical Research and the Norwegian Data Inspectorate approved the study. The cohort now includes more than 108.000 children. Questionnaire data were available for 66.808 children at 18 months, 51.447 children at 36 months and 14.181 children at 7 years of age. Pregnant women were recruited into MoBa until December 2008 and at the time of this study a large number of children had not yet reached 7 years of age. A total of 9 490 responded to the questionnaire at all the following three time points; 18 months, 36 months and 7 years. Of these, 463 respondents received a shortened questionnaire without dietary questions at age 36 months, in an attempt to raise the response rate by shortening the questionnaire and leaving out the dietary questions, resulting in n = 9 027. Gender information was available for 9 025 children; this sample is used in the present paper.

Intakes of fruit (excluding pure fruit juice), vegetables and SSB (including carbonated sugar-sweetened soft drinks and cordials (defined as sugar-sweetened concentrates of fruit and berries)) were assessed by frequency reported by a parent. The dietary questions were slightly different and the frequencies varied at the different time points due to several reasons. The inclusion of participants in MoBa started before all questionnaires were planned, and the follow-up questionnaires were developed one by one as funding was obtained. The overall aim was to include questions on as many exposures and health outcomes and developmental milestones of the child as possible. To make the most out of the available data, all variables were recoded into frequency of intake per week by using the midpoints of the categories (e.g. 1–2 times a week equal 1.5 times per week). As there is no consensus on how to deal with upper open-ended options, the lowest value of the upper open-ended options was used; such as 7 for "At least once a day" and 35 for "5 or more times in 24 hours" when reporting on a weekly basis. Finally, the frequencies were summed and categorized into three groups (times per week) based on what was possible across the time points. The specific questions and categories for frequency intakes of fruit, vegetables and SSB, as presented in the original questionnaires, are outlined in detail in Additional file 1: Table S1.

For fruit the recoded frequencies were summed and categorized into three groups; ≤ 5 times per week, 5.1-13.9 times per week and ≥ 14 times per week (representing those eating fruit two times per day on average, as recommended in the dietary guidelines). For vegetables the recoded frequencies were summed and categorized into three groups; ≤ 5 times per week, 5.1-7 times per week and > 7 times per week (representing those eating vegetables at least once a day on average). Finally, the recoded frequencies for SSB were summed and categorized into three groups; ≤ 1.5 times/glasses per week, 1.6-4.9 times/glasses per week and ≥ 5 times/glasses per week.

In MoBa, women answered questions related to health, education, income and lifestyle in a general questionnaire in early pregnancy (gestational week 15). Length of maternal education was calculated based on seven alternative answers for completed and ongoing education and divided into three categories; ≤ 12 years (high school or less), 13–16 years (4 years of university or university college), ≥ 17 years (more than 4 years of university).

### Data analysis

**Figure 1:**
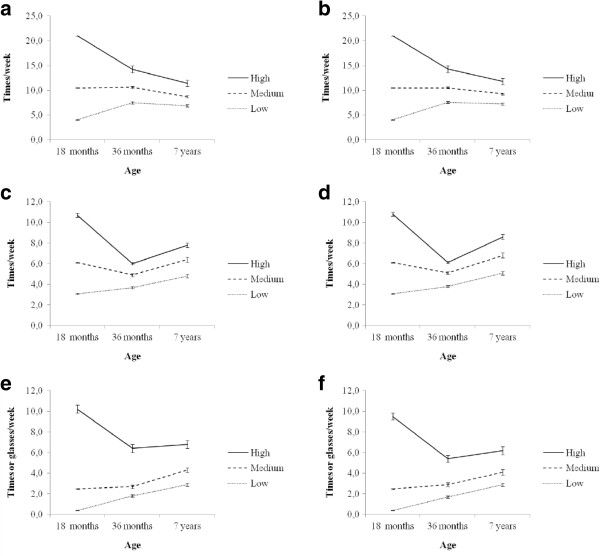
**Tracking patterns of fruit intake, vegetable intake and sugar-sweetened beverages (SSB) intake in boys (a, c and e) and girls (b, d and f), showing the intake frequencies of the children assigned to low, medium and high at 18 months, at ages 36 months and 7 years.** Low = low intake at 18 months, medium = medium intake at 18 months and high = high intake at 18 months.

The intakes of fruit, vegetables and SSB were not normally distributed and the numbers of subjects reported for the analyses vary due to missing values. For the analyses conducted, the data was categorized into three groups. Several methods have been used to describe the tracking of dietary behaviours over time. Firstly, proportions of children’s stability and change in dietary behaviours by gender from 18 months to 36 months of age, and from 18 months to 7 years of age were generated by cross-tabulation. Stability is shown by the percentage of individuals remaining in the same group of consumption at the time points and changes are presented by percentages of decrease or increase in consumption over time. Secondly, tracking coefficients of Spearman’s rho were calculated to test the correlation between each individual’s relative position in rank from 18 months to 36 months and from 18 months to 7 years of age. Cut-offs for the interpretation of Spearman’s rho are based on the range of values adopted in previous studies [34]. Thirdly, Figure 1 (a-f) show the intake frequencies of the children (assigned to low, medium and high at 18 months) at ages 36 months and 7 years.

Dietary behaviours at 18 months by gender and level of maternal education were generated by cross-tabulation and tested using the Chi-squared test. Additionally, multinomial logistic regression analyses were conducted to assess differences in the low and high consumption groups between children of mothers with low education versus high education. Multinomial logistic regression analyses were also used to evaluate the influence of maternal education (independent variable) on tracking of each of the dietary variables between 18 months and 7 years of age. A low level of education was considered a reference for the independent variable, and the stable intake was chosen as a reference in the dependent variable. All statistical analyses were performed by IBM® PASW® Statistics, version 18.0 (IBM Corp., Somers, New York, USA). The significance level was set to P < 0.05.

## Results

The median intake frequency of fruit for both genders was 7–10 times per week for all three age groups (Table 1). No significant differences were detected between intake of fruit at 18 months and 7 years of age among girls (P = 0.23), while all other differences were significant (P < 0.001). The median intake frequency of vegetables for both genders was 5–6 times per week for all three age groups. For intake of vegetables the differences were significant (P ≤ 0.02). The median intake frequency of SSB for both genders increased from 18 months to 7 years of age. The differences between the time points were significant for both genders (P < 0.001), except between 18 months and 36 months (boys P = 0.36, girls P = 0.23). Gender differences of importance in a public health perspective were found at 7 years of age, and for girls’ intake of SSB at age 18 months. Girls ate fruit and vegetables more frequently (at 7 years) and consumed SSB less frequently (at 18 months) compared to boys.

**Table 1:** Dietary intakes at age 18 months, 36 months and 7 years by gender

**Dietary intake**		**18 months**		**36 months**		**7 years**	
	**n**	**Median**	**5th–95th**	**Median**	**5th–95th**	**Median**	**5th–95th**
			**percentile**		**percentile**		**percentile**
**Boys**							
Fruit (times/week)	4484	10.5	(2.0–21.0)	7.0	(2.0–21.0)	7.5	(1.5–18.0)
Vegetables (times/week)	4507	6.0	(1.5–14.5)	5.0	(0.3–9.0)	5.5	(0.5–14.0)
SSB (times or glasses/week)	4374	2.0	(0–11.0)	2.0	(0.5–14.0)	2.5	(0–16.0)
**Girls**							
Fruit (times/week)	4247	10.5	(2.0–21.0)	7.0	(2.0–21.0)	8.0	(2.0–18.0)
Vegetables (times/week)	4289	6.0	(1.5–15.0)	5.0	(0.8–9.0)	6.0	(0.5–15.5)
SSB (times or glasses/week)	4143	1.0	(0–11.0)	2.0	(0.5-7.0)	2.5	(0–16.0)

*SSB* Sugar-sweetened beverages.

Differences between time points were significant except for:

- girls: fruit intake between 18 months and 7 years (P = 0.23).

- both genders: SSB intake between 18 months and 36 months (boys P = 0.36, girls P = 0.23).

Information about maternal education was available for 8686 (96%) of the participants, of which 34% were categorized at the low level (≤ 12 years), 46% at the medium level (13–16 years) and 20% at the high level (≥ 17 years). Comparison of children in the present study sample with all children in the 18 months sample showed significant but small differences in the characteristics of the dietary behaviours and the maternal education, indicating similar samples (Additional file 2: Table S2). For the included sample (n = 9025) the numbers of missing were low for the different dietary behaviours at age 18 months; fruit = 1.1% missing, vegetables = 0.5% missing and SSB = 2.8%. When comparing the children with data at age 18 months with those without data, by gender and maternal education, no differences were found for gender. There was a higher proportion of low educated mothers among those children with no data for fruit and SSB. No significant differences were detected for intake of vegetables (data not shown).

Tables 2 and 3 present the proportion of individuals’ stability in dietary behaviours from 18 months to 36 months of age, and from 18 months to 7 years of age, based on the groups of low, medium and high consumption. Largest changes were observed for the intake of fruit and SSB for both genders; more than 30% of the individuals increased their intake from 18 months to 7 years of age. Moreover, 50% of the boys and girls increased their intake of fruit from 18 months to 36 months, and a decrease in intake of vegetables was seen for 40% in the same period. Fair to moderate tracking coefficients (Spearman’s rho = 0.23-0.46) for the intake of fruit, vegetables and SSB were found from 18 months to 36 months and from 18 months to 7 years. From 36 months to 7 years the coefficients for boys were 0.32 (fruit), 0.40 (vegetables) and 0.39 (SSB) and for girls 0.29 (fruit), 0.43 (vegetables) and 0.38 (SSB).

**Table 2:** Proportion of stability and tracking coefficients in boys (n=4625)

**Dietary behaviours**	**18 months**				**36 months**				**7 years**			
	**n**	**%**	**Median**	**5th–95th**	**Decrease**	**Stability**	**Increase**	**Spearman’s rho**	**Decrease**	**Stability**	**Increase**	**Spearman’s rho**
				**percentile**	**%**	**%**	**%**		**%**	**%**	**%**	
**Fruit (times/week)**	**4484**				**8.8**	**40.8**	**50.3**	**0.36**	**18.3**	**51.5**	**30.2**	**0.23**
Low (≤ 5)	1659	37.0	5.0	(0.5–5.0)	n.c	37.6	62.4		n.c	42.2	57.8	
Medium (5.1–13.9)	2535	56.5	10.5	(10.5–10.5)	12.8	39.0	48.2		24.7	59.8	15.5	
High (≥ 14)	290	6.5	21.0	(21.0–21.0)	24.8	75.2	n.c		67.9	32.1	n.c	
**Vegetables (times/week)**	**4507**				**40.2**	**47.1**	**12.7**	**0.36**	**28.9**	**47.8**	**23.3**	**0.28**
Low (≤ 5)	1783	39.6	3.0	(0.5–5.0)	n.c	75.3	24.7		n.c	63.0	37.0	
Medium (5.1–7)	1075	23.9	6.0	(5.5–7.0)	55.1	32.7	12.3		42.6	21.2	36.2	
High (> 7)	1649	36.6	10.0	(7.5–17.5)	74.0	26.0	n.c		51.2	48.8	n.c	
**SSB (times or glasses/week)**	**4374**				**17.5**	**54.9**	**27.5**	**0.46**	**18.9**	**45.8**	**35.3**	**0.32**
Low (≤ 1.5)	2114	48.3	0.5	(0–1.0)	n.c	55.2	44.8		n.c	41.8	58.2	
Medium (1.6–4.9)	1171	26.8	2.5	(2.0–4.0)	25.6	52.3	22.0		24.9	48.2	26.9	
High (≥ 5)	1089	24.9	10.5	(5.0–25.0)	42.8	57.2	n.c		49.1	50.9	n.c	

*SSB* Sugar-sweetened beverages, *n.c.* no change (decrease/increase) of behaviour possible.

**Table 3:** Proportion of stability and tracking coefficients in girls (n=4400)

**Dietary behaviours**	**18 months**				**36 months**				**7 years**			
	**n**	**%**	**Median**	**5th–95th**	**Decrease**	**Stability**	**Increase**	**Spearman’s rho**	**Decrease**	**Stability**	**Increase**	**Spearman’s rho**
				**percentile**	**%**	**%**	**%**		**%**	**%**	**%**	
**Fruit (times/week)**	**4247**				**8.1**	**41.6**	**50.3**	**0.36**	**15.6**	**51.4**	**33.0**	**0.24**
Low (≤ 5)	1601	37.7	5.0	(2.0–5.0)	n.c	36.3	63.7		n.c	38.4	61.6	
Medium (5.1–13.9)	2375	55.9	10.5	(10.5–10.5)	11.8	41.3	46.9		20.3	62.3	17.4	
High (≥ 14)	271	6.4	21.0	(21.0–21.0)	23.6	76.4	n.c		66.8	33.2	n.c	
**Vegetables (times/week)**	**4289**				**39.8**	**47.1**	**13.1**	**0.37**	**26.0**	**48.9**	**25.2**	**0.31**
Low (≤ 5)	1677	39.1	3.0	(0.5–5.0)	n.c	73.3	26.7		n.c	59.8	40.2	
Medium (5.1–7)	1012	23.6	6.0	(5.5–7.0)	54.2	34.3	11.5		40.5	19.5	40.0	
High (> 7)	1600	37.3	10.0	(7.5–21.0)	72.3	27.7	n.c		43.9	56.1	n.c	
**SSB (times or glasses/week)**	**4143**				**17.0**	**54.3**	**28.8**	**0.44**	**19.9**	**45.1**	**35.0**	**0.30**
Low (≤ 1.5)	2111	51.0	0.5	(0–1.0)	n.c	55.6	44.4		n.c	44.0	56.0	
Medium (1.6–4.9)	1087	26.2	2.5	(2.0–4.0)	24.9	51.7	23.4		27.0	48.3	24.7	
High (≥ 5)	945	22.8	10.0	(5.0–24.5)	45.7	54.3	n.c		56.0	44.0	n.c	

*SSB* Sugar-sweetened beverages, *n.c.* no change (decrease/increase) of behaviour possible.

Tracking patterns of the children assigned to low, medium and high at 18 months, at ages 36 months and 7 years are shown in Figure 1(a-f). Although many children migrated between consumption groups, the intakes of the group originally classified as e.g. low continued to be low at age 36 months and 7 years, with no overlap seen between the intakes of any of the original consumption groups at subsequent time points. A tendency of regression towards the mean was generally observed, as group intakes tended to converge towards the centre distribution. A drop in intakes of the food groups was observed at the age of 36 months among the high consumers, and the drop was most pronounced for vegetables. The confidence intervals for the three groups (low, medium and high consumers) at the three time points were small for both genders and all the behaviours.

Differences in dietary behaviours at 18 months by gender and level of maternal education were significant (Table 4). Children of mothers with low education were more likely to consume fruit (P < 0.001, both genders) and vegetables (P < 0.001 for boys, P = 0.001 for girls) less often compared to children of mothers with high education. The reverse was found for consumption of SSB (P < 0.001, both genders).

**Table 4:** Dietary behaviors by gender and level of maternal education at 18 months of age

	**Boys**				**Girls**			
**Dietary behaviours at 18 months of age**	**Maternal education**							
	**≤ 12 years**	**13–16 years**	**≥ 17 years**		**≤ 12 years**	**13–16 years**	**≥ 17 years**	
				**P**				**P**
	**%**	**%**	**%**		**%**	**%**	**%**	
**Fruit (times/week)**								
Low (≤ 5)	48.0	34.2	25.9	< 0.001	48.4	34.0	28.0	< 0.001
Medium (5.1–13.9)	46.9	59.5	64.9		47.1	59.8	61.9	
High (≥ 14)	5.1	6.3	9.2		4.5	6.2	10.0	
**n = 4389 boys/4157 girls**	1448	2063	878		1445	1874	838	
**Vegetables (times/week)**								
Low (≤ 5)	42.3	39.1	34.8	< 0.001	42.2	38.1	36.1	0.008
Medium (5.1–7)	25.4	23.9	21.9		23.7	23.6	23.0	
High (> 7)	32.4	37.0	43.3		34.1	38.3	41.0	
**n = 4413 boys/4199 girls**	1459	2064	890		1467	1892	840	
**SSB (times or glasses/week)**								
Low (≤ 1.5)	42.8	49.8	54.1	< 0.001	45.1	51.8	59.3	< 0.001
Medium (1.6–4.9)	24.9	28.0	26.9		25.9	25.9	27.1	
High (≥ 5)	32.3	22.3	19.0		29.0	22.3	13.6	
**n = 4284 boys/4053 girls**	1404	2021	859		1409	1833	811	

*SSB* Sugar-sweetened beverages.

P from Chi-squared test.

Table 5 presents associations between maternal education level and whether parents reported their child to decrease or increase dietary intake compared with a stable intake from 18 months to 7 years of age. Boys and girls with mothers of high education had lower odds for an increase in intake of fruit (OR = 0.53, CI 0.44-0.65 and OR = 0.69, CI 0.57-0.84 respectively) and a decrease in intake of SSB (OR = 0.73, CI 0.58-0.92 in boys and OR = 0.61, CI 0.48-0.77 in girls), when compared to those with a stable intake having mothers of low education.

**Table 5:** Associations between dietary tracking from 18 months to 7 years and level of maternal education

**Dietary tracking**	**Boys (n = 4625)**			**Girls (n = 4400)**		
	**ME high versus ME low**			**ME high versus ME low**		
	**n**	**OR**	**CI (95%)**	**n**	**OR**	**CI (95%)**
**Fruit**	4389			4157		
Decrease	804	1.17	(0.93–1.47)	649	1.04	(0.81–1.33)
Increase	1327	0.53***	(0.44–0.65)	1372	0.69***	(0.57–0.84)
Stable	2258	1		2136	1	
**Vegetables**	4413			4199		
Decrease	1282	1.01	(0.83–1.23)	1095	0.84	(0.68–1.04)
Increase	1020	1.07	(0.87–1.32)	1060	1.04	(0.85–1.28)
Stable	2111	1		2044	1	
**SSB**	4284			4053		
Decrease	812	0.73**	(0.58–0.92)	802	0.61***	(0.48–0.77)
Increase	1516	0.92	(0.76–1.11)	1411	0.84	(0.69–1.02)
Stable	1956	1		1840	1	

*SSB* Sugar-sweetened beverages, *ME* Maternal education.

*** P < .001.

** P < .01.

For boys and girls with mothers of 13–16 years of education, only lower odds for an increase in intake of fruit were significant when compared to those with a stable intake having mothers of low education (data not shown).

## Discussion

We studied the changes and tracking in intakes of fruit, vegetables and SSB in a group of Norwegian children at three time points (18 months, 36 months and 7 years), and examined the association between maternal education and dietary tracking in the same group. The largest changes were seen for the intake of fruit and SSB from age 18 months to 7 years of age, while fair to moderate tracking coefficients for the intake of fruit, vegetables and SSB were found. Close to 50% of the children assigned to low, medium and high frequency of consumption at 18 months remained in the same group at age 36 months and 7 years. Children of mothers with low education were more likely to have a less frequent consumption of fruit and vegetables and a more frequent consumption of SSB, compared to children of mothers with high education at 18 months of age. Children of mothers with a high education level had lower odds for increasing fruit intake or decreasing SSB intake compared to children with a stable intake having mothers with a lower education level.

Comparison of children in the present study sample with all children in the 18 months sample showed significant differences for the dietary behaviours and maternal education. The level of maternal education in Norway has increased during the recruitment period (1999–2008) [35]. Moreover, healthier eating habits among preschool children have been associated with higher maternal education [18-24]. Taken together, this may explain the differences in the characteristics of the dietary behaviours and the maternal education.

The median intake frequencies of fruit and vegetables for both genders were below the national recommendations for all time points, while the consumption frequency of SSB was relatively low. Relative high proportions of stability were seen for the intakes of SSB in both genders, as found among Norwegian 11–13 year olds as well [36]. From 18 months to 36 months, 50% of the boys and girls increased their intake of fruit and 40% decreased their intake of vegetables. These findings may be affected by the number of questions and different frequencies used in the questionnaires [37]. In the present study there was one question for fruit at both 18 months of age and 36 months of age, but the frequency was higher at 36 months. This may have resulted in an overestimation of intake at 36 months of age (compared to 18 months of age), meaning that the proportion of children that actually increased their intake of fruit between the two time points is a bit lower than 50%. The same could be the case for the intake of vegetables, but in the opposite direction. The number of questions about vegetables was reduced from three to two between 18 months of age and 36 months of age, and the frequency was lower at 36 months of age. This may have resulted in an underestimation of intake at 36 months of age (compared to 18 months of age), meaning that the proportion of children that actually decreased their intake of vegetables between the two time points is a bit lower that 40%. However, our findings for the changes between 18 months and 36 months/7 years of age are in line with other studies that report percentages around 50% of children who remained in the same percentile/group during a certain period [34].

The number of studies investigating tracking in the childhood period is limited. Our results from Norway are in line with the findings in previous studies, even if they are not directly comparable; fair to moderate tracking was found both from 18 months to 36 months and from 18 months to 7 years. Stein *et al*. [28] reported fair to moderate tracking, as estimated by agreement of classification within quintiles of intake, of energy, fat, cholesterol, protein, carbohydrates, sodium, potassium and calcium in 3–5 year old children over a 19 month period of follow-up. Boulton *et al*. [29] found that tracking of energy and fat intake became more stable from 2 years of age in 106 children recruited at birth and followed prospectively until 15 years of age. Children with large energy intakes remained big eaters while children with low food intake became evenly spread across the distribution curve over time. Singer *et al*. [30] followed 95 children for 6 years, covering three age periods (3–4 years, 5–6 years and 7–8 years), and concluded that tracking of nutrient intake begins as young as 3–4 years of age. Moreover, extreme intakes tended to sustain over time. Robinson *et al.*[22] reported tracking of dietary patterns characterised as “infant guidelines” (high consumption of fruit, vegetables and home prepared foods) and “adult foods” (high consumption of bread, snacks, biscuits and chips) between children at 6 and 12 months of age, suggesting stability in eating habits that persist beyond infancy. When assessing the stability of dietary patterns like “processed”, “traditional” and “health conscious” in children at 3, 4, 7 and 9 years of age, Northstone and Emmet [31] found weighted ĸ for quintiles of dietary pattern scores in the range of 0.31 and 0.38 between 3 and 7 years of age. Moreover, the finding that children assigned to low, medium and high frequency of consumption stayed in the same group over time has also been found among Norwegian 11–13 year olds [36] and 14–21 year olds [38]. Finally, Boulton *et al*. [29] found a similar pattern and level of tracking among males and females in intake of energy, fat and calcium intake in 2–15 year olds.

Gender differences in frequency among the 7 year olds were observed in our sample for fruit, vegetables and SSB. No significant differences were found between boys and girls in intake of fruit, vegetables and SSB among the 4 year olds in the Norwegian Ungkost study from 2000 [39]. Gender differences in consumption of fruit, vegetables and SSB were larger in samples of Norwegian 10–13 years olds [14,15] indicating that girls start to eat healthier than boys already during early childhood. Additionally, previous research has found that girls compared to boys have a greater liking for and consumption of fruits and vegetables, while boys give higher ratings to fatty and sugary foods [40].

An important factor related to children’s dietary habits is maternal education. Results from previous studies are in concordance with our findings, suggesting that children of mothers with low education consumed fruit and vegetables less often and SSB more often compared to children of mothers with high education at 18 months of age. Several European studies of young children (from 6 months of age) have reported that lower maternal education is associated with a less healthy diet in the children compared to children who have mothers with higher education [18-24,39]. Finally, Vereecken *et al*. [41] found differences by educational level in children's and mothers' consumption frequencies of fruit, vegetables and soft drinks, and in the use of restrictions, verbal praise, negotiation, discouragement of sweets and restraining from negative modelling behaviour. Differences in children's food consumption by mothers' educational level were completely explained by mother's consumption and other food parenting practices for fruit and vegetables but not for soft drinks.

Finding that children of mothers with a high education level had lower odds for increasing fruit intake or decreasing SSB intake over time may be a consequence of limited possibilities for change, due to an already high consumption of fruit and a less frequent intake of SSB compared to those with a stable intake and having mothers of low education. Bere *et al*. [42] have reported that Norwegian adolescents of parents with higher education had a higher intake of fruit and vegetables, greater access to and preference for fruit and vegetables, greater knowledge of national recommendations, stronger intentions to eat 5-a-day and stronger role models. Their results support our findings. According to our study, the potential for improved intake of vegetables seems to be the same regardless of maternal education level.

In a public health perspective, our results indicate two main challenges; how to improve dietary habits among children of mothers with low education and how to maintain healthy dietary behaviours in children of mothers with high education during early childhood. Targeting mothers, and first-time mothers in particular, in nutrition education interventions has the potential to impact the dietary behaviours of young children indirectly and the mothers’ diet directly [25,26]. The interventions should target nutrition knowledge, attitudes, strategies and increase the awareness related to role modelling, regulation of unhealthy dietary habits and encouragement of healthy dietary behaviours [25,26,41].

Strengths of this study were the longitudinal study design based on a large national representative sample of children, and the use of multiple methods to describe tracking patterns over time. Both healthy and unhealthy dietary behaviours were studied, giving the opportunity to look at different dietary behaviours of young children. The main limitation of the study is the different variables and frequencies used at each time point to collect information about intake. The variances in intakes are small due to the large sample and the categories used. Analyses revealed only minor differences in the dietary behaviours at 18 months of age and maternal educational levels between the included children and the total sample at 18 months of age. Additionally, those with the most unhealthy dietary behaviours and lowest education were well represented in the sample included in the present study.

Finally, regression towards the mean as observed in the analyses presented in Figure 1, showed a decrease in frequencies among high consumers. This is the phenomena whereby the same variable is measured on two or more occasions, cases that are extreme on the first occasion will be somewhat less extreme on the second and third occasion [43].

## Conclusion

In this study we found that gender differences in dietary behaviours developed between 36 months and 7 years of age, suggesting that girls start to eat healthier than boys during early childhood. Furthermore, fair to moderate tracking coefficients for the intake of fruit, vegetables and SSB were found and children assigned to low, medium and high frequency of consumption at 18 months continued to be in the same group at age 36 months and 7 years. Children of mothers with low education were more likely to have a less frequent consumption of fruit and vegetables and a more frequent consumption of SSB, compared to children of mothers with high education at 18 months of age. Children of mothers with a high education level had lower odds for increasing fruit intake or decreasing SSB intake compared to children with a stable intake having mothers with a lower education level. Promotion of healthy dietary behaviours at an early age is important to prevent the establishment of unfavourable dietary behaviours later in childhood. Moreover, it seems important to target mothers in nutrition interventions for improving dietary habits among children, by teaching mothers nutrition knowledge, attitudes, strategies and increase the awareness related to role modelling, regulation of unhealthy dietary habits and encouragement of healthy dietary behaviours.

## Abbreviations

SSB: Sugar-sweetened beverages.

## Competing interests

The authors have no competing interests.

## Authors’ contribution

MB conducted the statistical analyses, wrote the first draft of the manuscript and made the greatest contribution to the paper. ALB and LFA collaborated closely in revising the manuscript. MH, HMM and WN participated in designing the study and/or in the final editing of the manuscript. All authors have critically revised the manuscript, and read and approved the final version.

## Pre-publication history

The pre-publication history for this paper can be accessed here:

http://www.biomedcentral.com/1471-2458/13/793/prepub

## Supplementary Material

Additional file 1: Table S1 Categories and frequencies in the different questionnaires used in the MoBa study. Click here for file

Additional file 2: Table S2 Characteristics for the total group at 18 months and those participating at three time points. Click here for file
