# Supplementary material for: Changes and tracking of fruit, vegetables and sugar-sweetened beverages intake from 18 months to 7 years in the Norwegian mother and child cohort study
Source: BMC Public Health. 2013 Aug 30;13:793. doi: 10.1186/1471-2458-13-793 (PMC3765981; doi:10.1186/1471-2458-13-793)
Supplement: Additional file 1: Table S1 — Categories and frequencies in the different questionnaires used in the MoBa study. [file 1471-2458-13-793-S1.pdf]

Additional file 1

Table S1 Categories and frequencies in the different questionnaires used in the MoBa study

**Questionnaire 5 - 18 months**
How often do you give your child the following to eat/drink now that he/she is 18 months old?
**Version 1 (n = 108)**<br>*Fruit (1 variable)*<br>*Vegetables (3 variables): Peas, beans - Other cooked vegetables - Raw vegetables*<br>*SSB (2 variables): Cordial, sweetened - Fizzy drinks*<br>FQ: Never/seldom, 1-3 times a week, 4-6 times a week, At least once a day
**Version 2 (n = 8197)**<br>*Fruit (1 variable)*<br>*Vegetables (3 variables): Peas, beans - Other cooked vegetables - Raw vegetables*<br>Never, Less than once a week, 1-3 times a week, 4-6 times a week, 1-2 times in 24 hours,<br>3 or more times in 24 hours<br>*SSB (2 variables): Cordial, sweetened - Fizzy drinks*<br>FQ: Never, Less than once a week, 1-3 times a week, 4-6 times a week, 1-2 times in 24 hours, 3-4 times in 24 hours, 5 or more times in 24 hours
**Questionnaire 6 - 36 months (n = 9025)**
How often does your child drink or eat the following at present?
Fruit (1 variable)<br>*SSB (1 variable)*<br>FQ: Seldom/less than once a week, 1-3 times a week, 4-6 times a week, Once in 24 hours, Twice in 24 hours, 3 times in 24 hours, 4 or more times in 24 hours

Vegetables (2 variables): Cooked vegetables - Raw vegetables, salad<br><br>FQ: Once a month or less often, 2-3 times a month, Once a week, Twice a week, 3 times a week, 4 times a week, 5 or more times a week
**Questionnaire 7 - 7 years (n = 9025)**
How often does your child usually drink/eat the following?
Fruit (4 variables): Oranges, tangerines - Apples, pears, grapes - Bananas - Other fresh fruit or berries<br><br>*Vegetables (4 variables): Carrots - Cabbage, cauliflower, broccoli - Salad - Other vegetables*<br><br>FQ: Never/seldom, 1-3 times a month, 1-2 times a week, 3-4 times a week, 5-6 times a week, Once a day or more often<br><br>*SSB (3 variables): Apple nectar, other nectar - Cordial, sweetened - Fizzy drinks*<br><br>FQ: FQ: Never, 1-3 glasses a month or less often, 1-3 glasses a week, 4-6 glasses a week,<br><br>1-3 glasses in 24 hours, 4 or more glasses in 24 hours

SSB: Sugar-sweetened beverages
