# Supplementary material for: Changes and tracking of fruit, vegetables and sugar-sweetened beverages intake from 18 months to 7 years in the Norwegian mother and child cohort study
Source: BMC Public Health. 2013 Aug 30;13:793. doi: 10.1186/1471-2458-13-793 (PMC3765981; doi:10.1186/1471-2458-13-793)
Supplement: Additional file 2: Table S2 — Characteristics for the total group at 18 months and those participating at three time points. [file 1471-2458-13-793-S2.pdf]

Additional file 2

Table S2 Characteristics for the total group at 18 months and those participating at three time points

	18 months			
**Dietary behaviours and maternal education**	Total (n = 57783)		Three time points (n = 9025)	
	n	%	n	%
**Fruit (times/week)**	56674		8926	
Low (≤ 5)	17513	**30.9**	3353	**37.6**
Medium (5.1-13.9)	34428	**60.7**	5002	**56.0**
High (≥ 14)	4733	**8.4**	571	**6.4**
**Vegetables (times/week)**	57263		8983	
Low (≤ 5)	21634	**37.8**	3545	**39.5**
Medium (5.1-7)	12820	**22.4**	2126	**23.7**
High (> 7)	22809	**39.8**	3312	**36.9**
**SSB (times or glasses/week)**	55540		8771	
Low (≤ 1.5)	34630	**62.4**	4357	**49.7**
Medium (1.6-4.9)	11931	**21.5**	2309	**26.3**
High (≥ 5)	8979	**16.2**	2105	**24.0**
**Maternal education**	54990		8686	
Low (≤ 12 years)	16689	**30.3**	2967	**34.2**
Medium (13-16 years)	23862	**43.4**	3985	**45.9**
High (≥ 17 years)	14439	**26.3**	1734	**20.0**

SSB: Sugar-sweetened beverages

All differences between the total sample and those participating at three time points are significant P<.001
